# Job preferences of primary health care workers in rural China: application of Discrete Choice Experiment

**DOI:** 10.1186/1472-6963-14-S2-P69

**Published:** 2014-07-07

**Authors:** Xiaoyun Liu

**Affiliations:** 1 China Center for Health Development Studies, Peking University, Beijing, China

## Background

The shortage of qualified health workers in primary health care (PHC) in rural China has been a long-term challenge in China. Health workers’ job preferences and job choices are determined by various external and intrinsic factors. These factors are constantly changing along with the health system reform. This study aims to quantitatively measure job preferences of health workers in rural areas in the context of health system reform in China.

## Materials and methods

A Discrete Choice Experiment (DCE) was applied to study the determinants of health workers’ job preferences. Using a multi-stage stratified sampling process, 228 doctors and 165 nurses were selected from 45 township health centers in 3 provinces in China. 6 job attributes were included in the DCE design (working location, income, permanent position, training opportunities, children’s education opportunities, and career development). Questionnaires with 16 questions were self-administered in August 2013. A conditional logit model was applied to analyze the DCE data.

## Results

Income level was the most important factor for health workers’ attraction and retention (OR=7.0 when comparing 8000 RMB versus 2000 RMB per month). The second important factor was education opportunities for their children (OR=4.3 for females and 2.9 for males). The third important factor was a permanent position (OR=2.7).

## Conclusions

Extrinsic factors play dominant role in attracting and retaining PHC workers in rural China. Government should take a leading role in response to the inequitable distribution of health workers, especially in resource poor settings where health workers’ income are far beyond a satisfactory level. Application of the DCE should be in close collaboration with relevant policy makers for the research to have policy engagement.

